# Revision of failed elbow internal joint stabilizer with hinged elbow external fixator and augmented ligament repair: a technical report

**DOI:** 10.1016/j.xrrt.2026.100864

**Published:** 2026-09-15

**Authors:** George Sayegh, Nicholas Phan, Adam Maestas, Anil K. Dutta

**Affiliations:** University of Texas Health San Antonio School of Medicine, San Antonio, TX, USA

**Keywords:** Internal joint stabilizer, Hinged external fixator, Recurrent elbow dislocation, Two-step approach, Posterolateral rotatory instability, Lateral ulnar collateral ligament repair

Posterolateral rotatory instability of the elbow is a complex problem arising when both static and dynamic stabilizers of the joint are compromised. The lateral ulnar collateral ligament (LUCL) serves as the primary soft-tissue restraint to varus and external rotatory stress, while the coronoid process provides critical bony buttress against posterior subluxation.^1^^,^^3^^,^^12^^,^^15^ Disruption of these structures, particularly when combined with osseous injury or failed fixation, renders the elbow unstable and predisposed to recurrent dislocation.^17^ Standard operative strategies, such as isolated ligament repair or fracture fixation, often fail to fully restore stability, especially in elderly patients with diminished bone quality or in revision scenarios after hardware failure.^8^^,^^20^

Hinged external fixation (HEF) has emerged as an important adjunct in these settings, offering a means of restoring stability while permitting early, guided range of motion. The device functions by replicating the elbow's native flexion-extension axis, offloading stress from repaired ligaments and fracture constructs, and minimizing the risk of recurrent dislocation.^4^ Successful use, however, requires a meticulous technique: biomechanical studies demonstrate that even a 5° deviation or 5-mm translation from the anatomic axis of rotation significantly increases joint constraint and impairs motion.^10^^,^^16^ When applied accurately, HEF allows for early mobilization, reduces post-operative stiffness, and serves as a “mechanical bridge” to biological healing of both bone and soft tissues. By restoring the native axis of rotation, the fixator reduces torsional stress on healing ligamentous and capsular repairs, which is particularly important in revision cases with compromised soft-tissue integrity. Clinical series and systematic reviews have reported favorable outcomes with HEF for complex instability, with improvements in functional scores and range of motion and acceptable complication rates, particularly when combined with ligamentous repair and fracture fixation.^4^^,^^17^ In the present case, we describe an elderly patient who sustained a traumatic elbow dislocation complicated by hardware failure of an internal joint stabilizer (IJS). Her revision surgery required a comprehensive approach: removal of failed hardware, open reduction, anatomic ligamentous repair, and application of a hinged external fixator in what we term the “San Antonio Two-Step” technique. To our knowledge, this specific stepwise sequence for intra-operative hinge alignment has not been previously described in the peer-reviewed literature. The “San Antonio Two-Step” represents a novel technique introduced by the senior authors that standardizes intra-operative hinge alignment through a reproducible sequential adjustment algorithm. This case highlights the indications, technical nuances, and clinical outcomes of HEF in the management of complex elbow instability following failed stabilization. We hypothesized that precise, reproducible hinge alignment using the San Antonio Two-Step, combined with anatomic ligament repair, would restore stable, pain-free motion without recurrent dislocation despite the patient's advanced age and compromised bone quality.

## Case description

**Figure 1:**
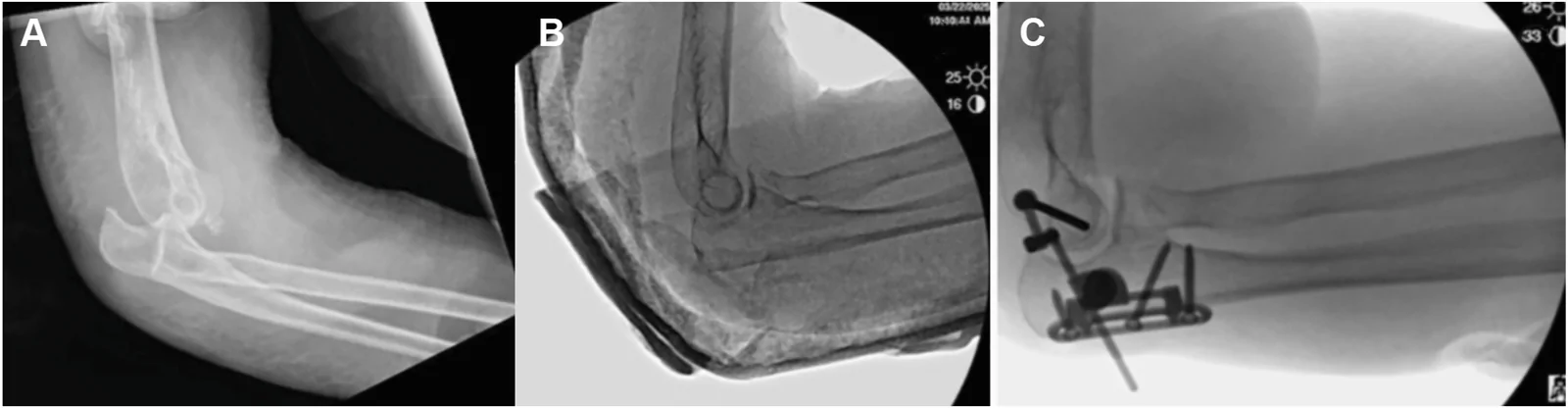
Radiographic imaging of the patient's right elbow. Initial imaging (**A**) demonstrates elbow dislocation following the fall. Reduction radiographic imaging (**B**) was obtained following reduction in the emergency room. Initial post-operative radiographic imaging (**C**) following open reduction and internal fixation with an internal joint stabilizer at an outside institution.

This is a technical case report describing revision surgical management of a single patient with a failed elbow IJS, combining anatomic ligament repair with HEF using a reproducible technique for hinge alignment. The patient was an 84-year-old right-hand-dominant female who presented to an outside institution after a fall in which she sustained a right elbow dislocation along with an ipsilateral distal radius fracture (Fig. 1, *A* and *C*). No associated coronoid or radial head fracture was identified. The injury pattern was that of a combined ligamentous (posterolateral rotatory and valgus) instability with an ipsilateral, closed distal radius fracture rather than a bony fracture-dislocation. The distal radius fracture was treated non-operatively in a brace. Non-operative management was elected given the patient's advanced age, functional demands, and fracture stability on serial radiographs, in the context of prioritizing surgical treatment of the more urgent, grossly unstable elbow injury. The elbow was reduced in the emergency room, and she underwent operative management with open reduction of the ulnohumeral joint with placement of an IJS (Fig. 1, *B* and *C*). The immediate post-operative course was uncomplicated, and she was placed into a protective splint.

**Figure 2:**
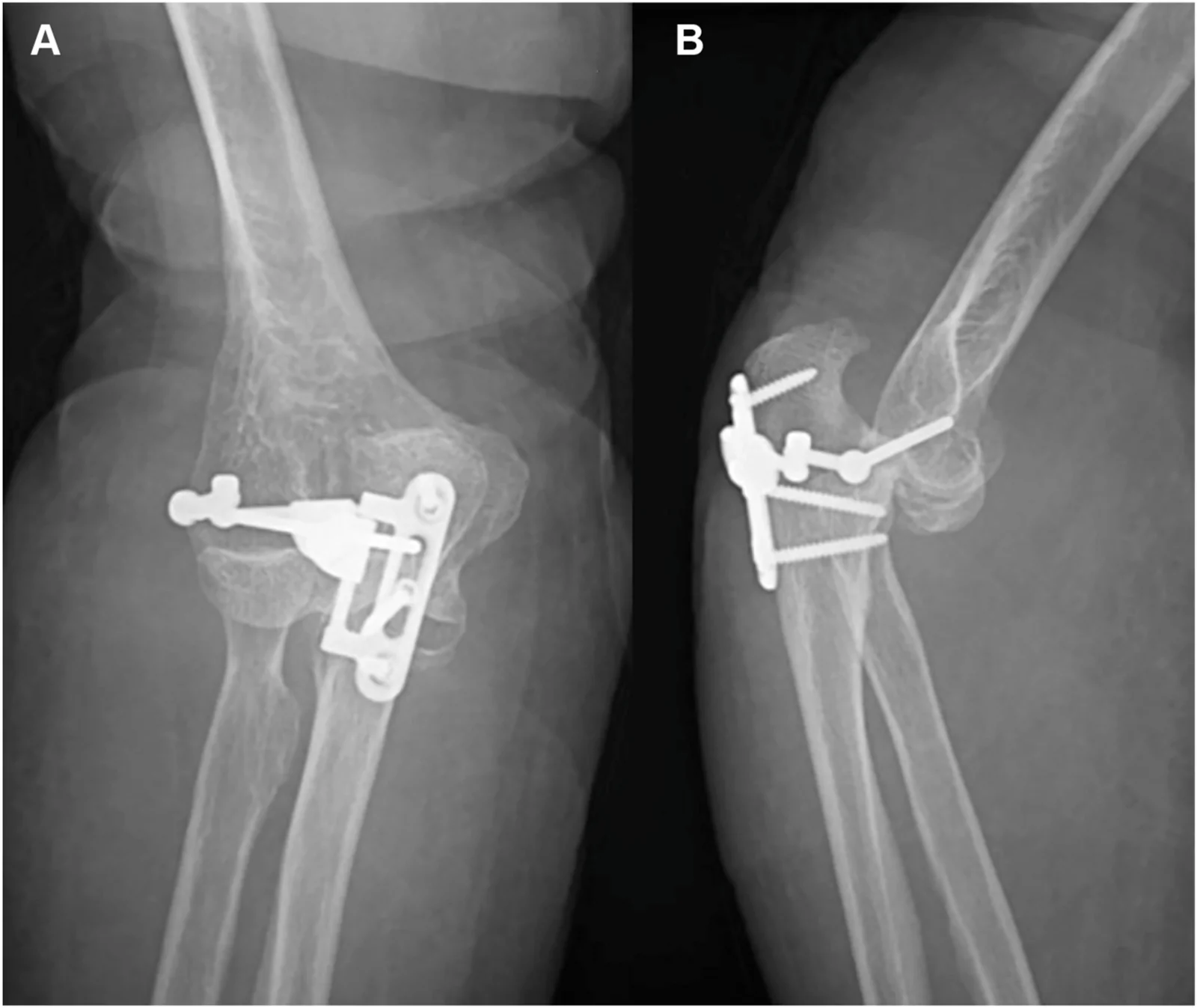
Antero-posterior (**A**) and lateral (**B**) radiographic imaging of recurrent elbow dislocation in the post-operative period of open reduction and internal fixation with an internal joint stabilizer.

At her follow-up visit 12 days later, she was found to have recurrent elbow dislocation, with radiographs demonstrating failure of the IJS hardware (Fig. 2, *A* and *B*). Clinical examination revealed swelling, deformity, and pain about the right elbow. Despite the gross instability, neurovascular function remained intact, with preserved motor strength in the median, ulnar, and radial distributions and palpable radial and ulnar pulses. The skin incision was well approximated without signs of infection. Although the precise mechanism of failure could not be definitively established, the absence of wound complications or infection at this visit, combined with the patient's advanced age and osteoporotic bone quality, suggests loss of fixation (hardware pullout) in compromised bone as the most probable etiology, rather than a discrete technical or compliance-related factor. At that time, the distal radius fracture continued to be managed conservatively.

Given the combination of recurrent instability, hardware failure, and her advanced age, revision surgery was indicated. Given that the IJS failure reflected inadequate fixation in osteoporotic juxta-articular bone, revision using a second internal hinge device carried a similar risk of recurrent loss of fixation. A hinged external fixator was therefore favored as it distributes load across multiple extra-articular fixator pins rather than relying on screw purchase in the same compromised bone stock. Five days later, she underwent a revision surgery at our institution. The operative plan included removal of the failed IJS, débridement of interposed tissue within the joint, reduction of the ulnohumeral articulation, ligamentous repair with particular attention to the LUCL, and application of a hinged external fixator to restore stability and allow early motion.

## Surgical technique

The patient was positioned supine on a standard operating table. A rolled sheet was placed under the operative shoulder to elevate the extremity. The table was tilted slightly into reverse Trendelenburg with the head elevated and rotated 90° to allow the C-arm to enter from the head of the bed. The operative extremity was positioned near the edge of the table to facilitate intra-operative imaging.

After the induction of general anesthesia, a sterile tourniquet was applied to the proximal arm. The extremity was prepared with alcohol and hydrogen peroxide, followed by DuraPrep (Solventum, Maplewood, MN, USA) and ChloraPrep (BD, Franklin Lakes, NJ, USA). Ioban (Solventum), stockinette (McKesson, Richmond, VA, USA), and Coban (Solventum) were applied distally, leaving the hand available for motor testing. Sticky U-drapes and split sheets were used to complete sterile field preparation.

### Incision and exposure

Critical landmarks were identified, including the medial and lateral epicondyles, the subcutaneous dorsal border of the proximal ulna, and the lateral column of the humerus. A posterior longitudinal incision was used. In this case, the prior lateral-based incision was incorporated and extended. The lateral column and joint were exposed through the Kocher interval between the anconeus and extensor carpi ulnaris. Medial column exposure was performed to aid reduction and remove interposed tissue. The ulnar nerve was identified and decompressed in situ. No anterior transposition was performed.

### Hardware removal and joint preparation

All IJS components were removed, including the plate and screws. To facilitate complete visualization and débridement of the ulnohumeral articulation, the elbow was intentionally re-dislocated. Pulvinar tissue and capsular scarring were excised, allowing restoration of a concentric reduction once the joint surfaces had been adequately prepared. Fluoroscopy confirmed congruent alignment of the ulnohumeral joint. The coronoid process and radial head were directly visualized during débridement and were found to be intact without a fracture or significant chondral injury. Varus and posterolateral rotatory stress testing under fluoroscopy prior to ligament repair confirmed gross instability of the ulnohumeral joint, establishing the baseline instability pattern addressed by the subsequent repair.

### Ligamentous repair

The LUCL origin was exposed at the lateral epicondyle. The footprint was prepared for the bleeding bone. A suture anchor was placed at the isometric point just posterior to the capitellum. Sutures were passed through the LUCL and tied to restore continuity to the supinator crest of the ulna. Stability was restored through direct repair of native ligamentous tissue augmented by suture anchor fixation.

After lateral repair, valgus stress testing demonstrated persistent instability. The anterior bundle of the medial ulnar collateral ligament (MUCL) was therefore repaired. The medial epicondyle footprint was prepared, and a suture anchor was placed. The ligament was tensioned in 30° of flexion to restore valgus stability. This medial-sided repair performed after intra-operative valgus stress testing following lateral repair revealed persistent instability, reflecting a stepwise algorithm for addressing combined instability rather than a pre-determined bilateral approach.

### Placement of the hinged external fixator

**Figure 3:**
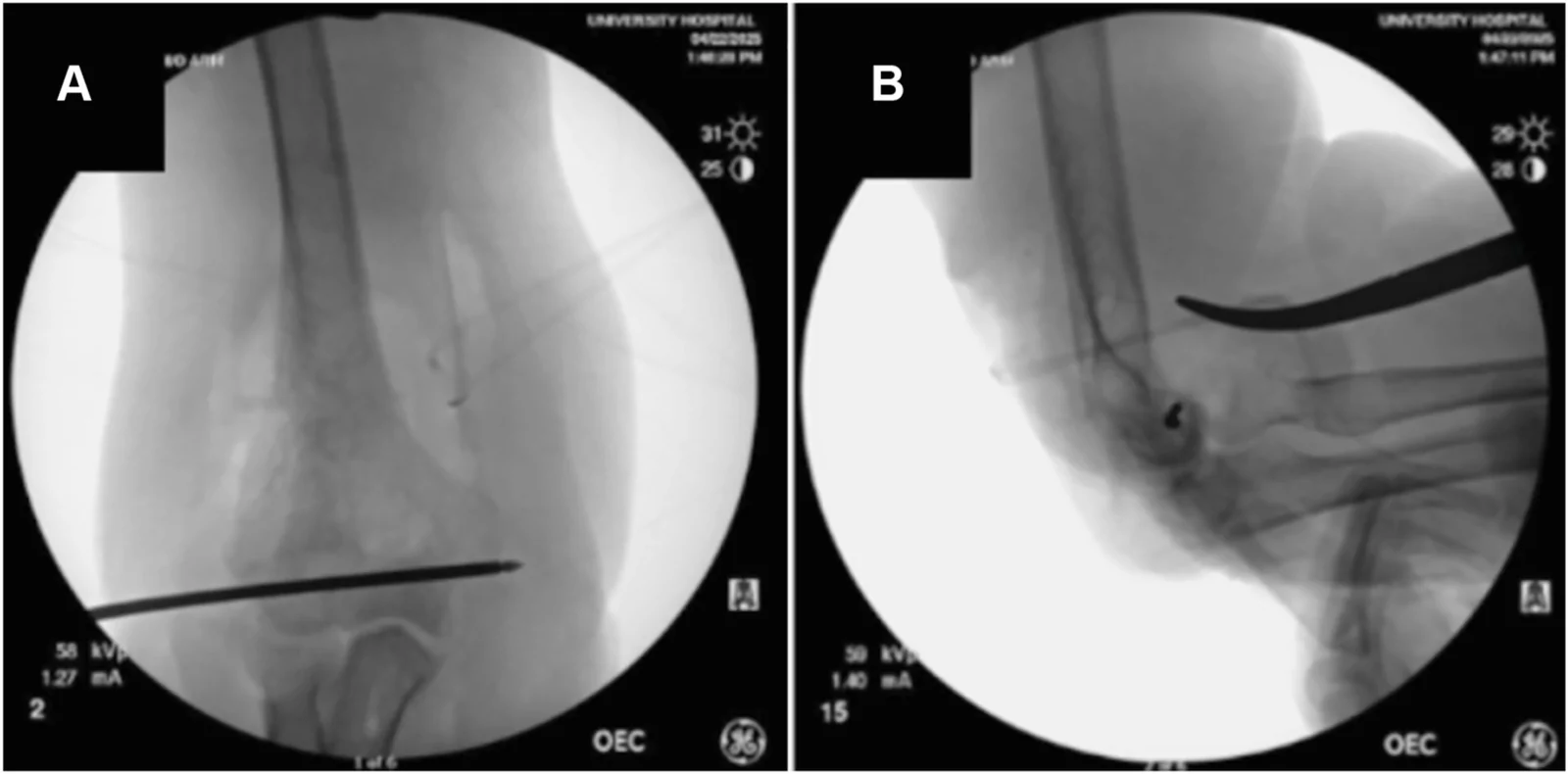
Antero-posterior (**A**) and lateral (**B**) intra-operative fluoroscopic imaging of the trans-trochlear flexion-extension axis.

The trans-trochlear flexion-extension axis was identified under fluoroscopy. An axis pin was inserted from the capitellar isometric point laterally to the center of the trochlea medially, just anterior and distal to the epicondyle (Fig. 3, *A* and *B*).

Schanz pins were then placed into the distal humerus and proximal ulna. Blunt dissection was used for the bone to avoid neurovascular injury, with particular attention to palpating and protecting the radial nerve during humeral pin placement. Self-drilling, self-tapping pins were placed bicortically, and fixator clamps were attached.

The elbow was reduced and coupled to the hinge. The alignment and securing of the hinged external fixator was finalized using the “San Antonio Two-Step” method. This approach ensures that the hinge axis is correctly balanced with the native flexion-extension arc of the elbow, minimizing mal-tracking and allowing for an early, safe motion.

#### Step 1 (flexion and provisional tightening)

The elbow is concentrically reduced in flexion. Pin-to-bar clamps are provisionally tightened beginning with those closest to the joint. This establishes preliminary stability while the elbow remains in a position of maximal bony congruency.

#### Step 2 (extension and final adjustment)

**Figure 4:**
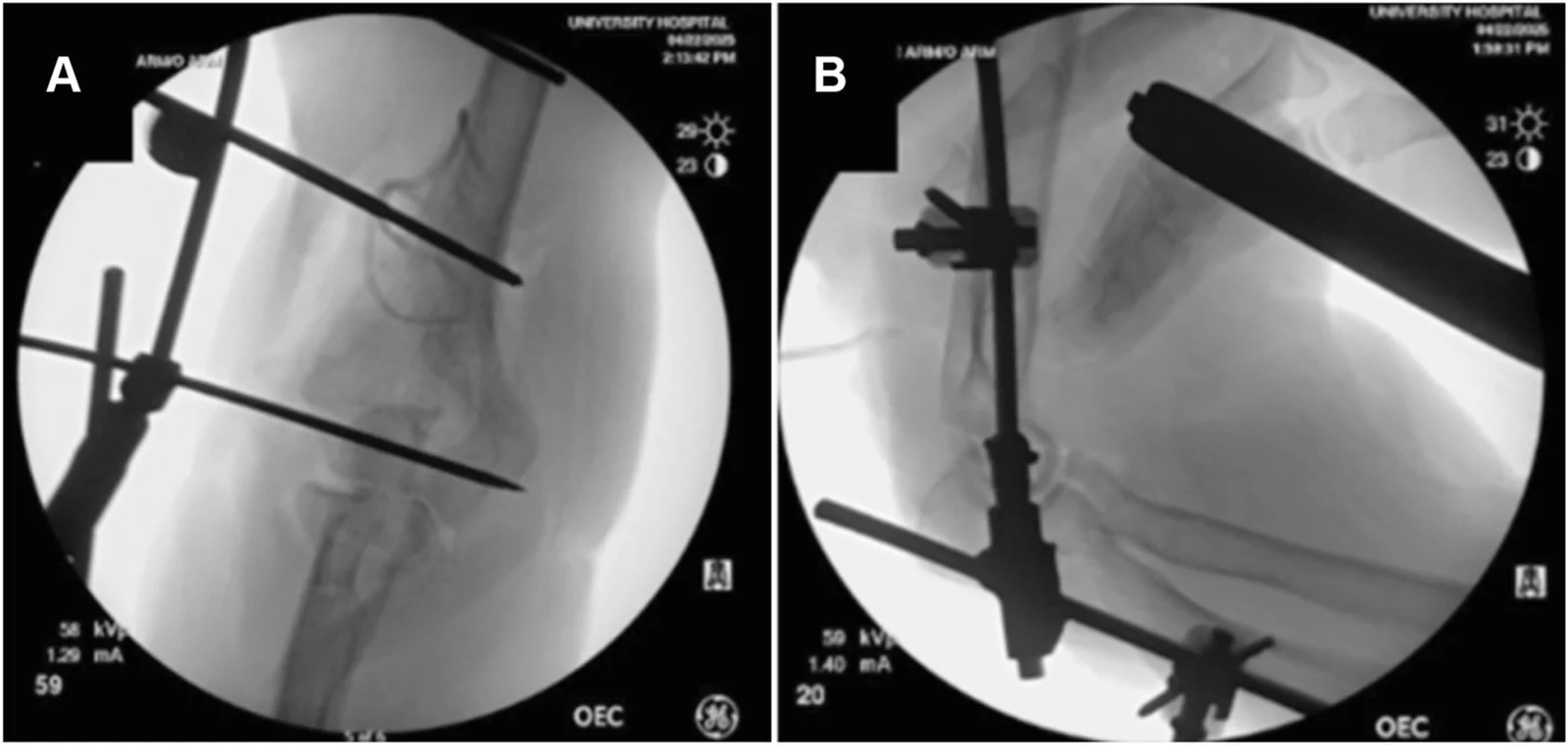
Antero-posterior (**A**) and lateral (**B**) intra-operative fluoroscopic imaging of proper reduction following hinged external fixation application.

The elbow is then gently extended under fluoroscopy (Fig. 4, *A* and *B*). Smooth tracking through the arc confirms appropriate hinge alignment. If mal-tracking is observed, clamps are sequentially loosened and re-tightened until concentric reduction is maintained throughout the full arc of motion. This process is repeated until the joint space remains concentric and symmetric throughout the arc, consistent with published tolerances of less than 5° of angular deviation and less than 5-mm translation from the native axis of rotation. Once alignment is confirmed, all connections are fully tightened. A temporary crossbar is then placed, usually maintaining the elbow in flexion for one to two weeks to provide added stability during the initial post-operative phase.

This reproducible two-step sequence allows reliable fine-tuning of hinge alignment and provides a structured way to achieve stable reduction. In our experience, it reduces variability, protects ligament and bony repairs, and permits early mobilization without recurrent subluxation.

### Closure and outcome

**Figure 5:**
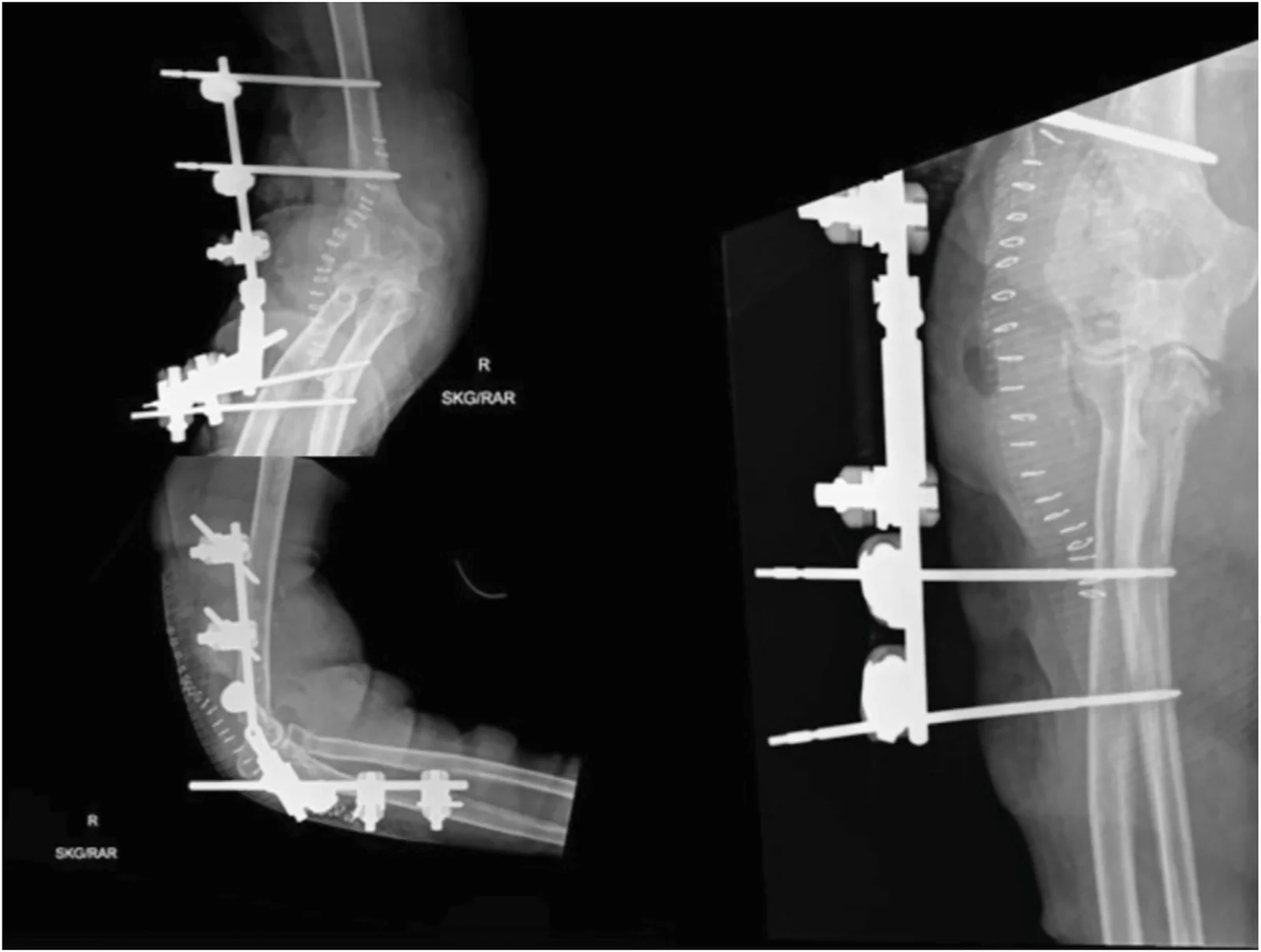
Final radiographic images of the patient's elbow post operation.

The wound was irrigated and closed in layers. Skin was closed with staples, and pin sites were dressed with Xeroform (Cardinal Health, Dublin, OH, USA) and Kerlix (Cardinal Health). Final x-rays confirmed concentric reduction of the ulnohumeral and radiocapitellar joints, with the hinged fixator intact (Fig. 5).

Post-operatively, she was immobilized in the hinged fixator with the elbow concentrically reduced. Her neurovascular status remained intact, with the ability to perform thumbs up, OK sign, and index-middle finger separation. The radial artery pulse was palpable. Early radiographs demonstrated satisfactory reduction with the fixator in place. Her post-operative plan included immobilization in the hinged frame, early passive- and active-assisted motion in a controlled arc, and serial follow-up visits for wound- and pin-site evaluation. She was followed up for approximately 6 weeks, with the hinged fixator maintained for 4-6 weeks to allow protected, progressive motion before removal. At the time of fixator removal, the elbow remained concentrically reduced without recurrent instability, and the patient had regained a functional arc of motion adequate for activities of daily living. Formal validated outcome instruments (eg, Mayo Elbow Performance Score and QuickDASH) and a numeric pain score were not systematically collected during this early follow-up period; this is acknowledged as a limitation below.

## Discussion

Revision of failed IJS procedures for elbow instability is technically demanding and most often required because of recurrent instability, hardware complications, or progressive post-traumatic degeneration.^9^ While the IJS has been shown to restore stability and permit early mobilization,^7^ recurrent instability and device-related complications are not uncommon, particularly in complex cases or revision settings.^19^

HEF provides an alternative means of dynamic stabilization. By restoring the native flexion-extension axis, HEF protects ligament and capsular repairs while allowing early range of motion. Several series have reported satisfactory functional outcomes, with most patients regaining a functional arc of motion and maintaining concentric reduction.^18^ However, device-related complications such as pin-tract infection, nerve injury, and hardware failure are more frequent with HEF than with simpler methods like cross-pinning, though long-term function and motion arc are often comparable.^5^

Restoration of concentric reduction after chronic or recurrent dislocation often requires removal of the interposed soft tissue.^2^^,^^22^ Pulvinar scar and capsular contracture have been shown to block reduction and contribute to persistent instability if not adequately addressed.^5^ Anderson et al^2^ similarly demonstrated that débridement of interposed pulvinar and capsular tissue, combined with hinged fixation, restored stable concentric reduction and permitted early motion in chronic elbow dislocation, supporting intentional re-dislocation and débridement as a necessary rather than incidental step in revision cases. Intentional re-dislocation and débridement, as performed here, is therefore not simply a technical maneuver but a necessary step to recreate a congruent articulation that can support ligament repair and long-term stability.

Stabilization of both the lateral and medial collateral ligament complexes was critical given the combined instability pattern. The LUCL is the primary restraint to posterolateral rotatory instability, and its deficiency alone is sufficient to produce recurrent subluxation.^14^ Similarly, the MUCL is the principal valgus stabilizer, particularly under functional loading.^13^ Prior studies have shown that isolated repair of one complex in the setting of global instability carries a high risk of recurrent laxity.^6^ Addressing both sides in this patient therefore provided a biologic basis for stability, aligning with current recommendations for complex revision scenarios.

The decision to perform primary repair or graft re-construction of the collateral ligaments in revision settings remains debated. A recent systematic review and meta-analysis found that LUCL re-construction was associated with a superior rate of return to activity and a lower complication rate than repair, although post-operative range of motion and patient-reported outcomes were similar between techniques.^11^ In the present case, direct repair of both the LUCL and MUCL was performed using suture anchor fixation of the native ligamentous tissue rather than graft re-construction, reflecting adequate quality of the native ligament tissue identified intra-operatively. We would favor re-construction with a tendon graft in cases where native tissue is of insufficient quality to support anchor-based repair, particularly in revision settings following a prior failed ligament surgery.

Accurate hinge placement with a hinged external fixator is equally important. Biomechanical studies demonstrate that even a 5° deviation or 5-mm translation from the true axis of rotation increases joint constraint and limits motion.^21^ This is particularly relevant in revision cases where stiff scarred tissue magnifies the consequences of mal-alignment. Existing descriptions of HEF emphasize accurate fluoroscopic localization of the elbow's flexion-extension axis and careful intra-operative assessment of joint tracking, but they generally rely on the surgeon’s experience for iterative clamp adjustment once provisional fixation has been achieved. In contrast, the San Antonio Two-Step procedure introduces a structured, reproducible sequence for hinge optimization by specifying the order of clamp adjustment, standardized fluoroscopic assessment through the arc of motion, and sequential correction of mal-tracking one fixation point at a time. Although this technique has not yet undergone formal biomechanical or cadaveric validation, we believe this systematic approach may reduce technical variability and facilitate reproducible hinge alignment, particularly in complex revision cases where accurate restoration of elbow kinematics is especially challenging. The San Antonio Two-Step procedure therefore provides a reproducible method to fine-tune axis placement, building on existing literature that emphasizes the need for precision in hinge alignment.^5^ In this way, the method does not simply supplement repairs but actively reduces the risk of re-dislocation and post-operative stiffness.

The patient achieved stable concentric reduction, had no recurrent dislocation, and regained a functional arc of motion. The distal radius fracture healed uneventfully with non-operative care. No device-related complications, including pin-tract infection, loss of fixation, or neurovascular compromise, were encountered. These outcomes highlight that even in elderly patients with failed prior fixation, a combined strategy of ligament repair and HEF with the San Antonio Two-Step procedure may represent a viable salvage strategy. These findings, however, are drawn from a single case and require validation in larger series before broader conclusions can be drawn.

This report is limited by its single-patient design and a relatively short (approximately 6 weeks) follow-up period. Formal, validated functional outcome instruments (eg, Mayo Elbow Performance Score and QuickDASH) and a numeric pain score were not systematically collected, limiting objective assessment of functional recovery. The San Antonio Two-Step technique has not undergone cadaveric or biomechanical validation, and its reproducibility and accuracy relative to other described methods of hinge alignment remain to be formally tested. Because this case was managed with conversion to HEF rather than revision IJS, no direct comparison between these 2 salvage strategies can be drawn from this report. As a single-patient technical report, these findings have limited external validity and generalizability, and larger comparative studies are needed to assess reproducibility, long-term outcomes, and a direct comparison to other salvage strategies such as revision IJS or total elbow arthroplasty. No author has a financial relationship with the manufacturer of the IJS or hinged external fixator devices used in this report, and no manufacturer funding was received for this work. Nevertheless, this case underscores the potential value of combining anatomic ligament repair with accurate, reproducible hinge alignment as a salvage option for complex elbow instability in selected patients.

## Conclusion

Revision stabilization of a failed IJS with augmented ligament repair and HEF using the San Antonio Two-Step technique may represent a useful salvage option in selected patients, protecting biologic repairs, restoring the native joint axis, and enabling early motion in complex cases of elbow instability.

## Disclaimers

Funding: No funding sources were received for this article.

Conflicts of interest: Dr. Anil K Dutta received royalties from Enovis and Zimmer Biomet and served as a consultant for Enovis and Zimmer Biomet. Any additional authors, their immediate families, and any research foundations with which they are affiliated have not received any financial payments or other benefits from any commercial entity related to the subject of this article.
